# Effect of fetuin-A on adenine-induced chronic kidney disease model in male rats

**DOI:** 10.22038/IJBMS.2023.66346.14584

**Published:** 2023

**Authors:** Wesam M.R Ashour, Mohamed Sayed Ahmed Zamzam, Heba Essam El Din El Sayed Ali, Reham Hassan Ebrahim

**Affiliations:** 1 Physiology Department, Faculty of Medicine, Zagazig university, Sharqia government, Egypt

**Keywords:** Adenine, Chronic renal failure, Fetuin-A, Inflammation, Kidney function tests

## Abstract

**Objective(s):**

This study aimed to investigate the possible effects of fetuin-A on an adenine-induced chronic kidney disease (CKD) model in male rats.

**Materials and Methods:**

Rats were divided into three groups: group A included rats fed a normal diet; group B included rats fed a normal diet with 220 mg/kg adenine daily for 21 days; group C included rats fed a normal diet with 220 mg/kg adenine daily for 21 days and intraperitoneally administered with 5 mg\kg fetuin-A every other day for 2 weeks. Serum samples were assayed for serum creatinine, urea, sodium, potassium, calcium, phosphorus, tumor necrosis factor (TNF), interleukin-6 (IL-6), and estimated glomerular filtration rate (eGFR), and immunohistochemical staining was performed.

**Results:**

Group B showed a significant increase in serum creatinine, urea, phosphorus, potassium, TNF, and IL-6 and a significant decrease in serum sodium, calcium, and eGFR compared with group A. Regarding immunohistochemistry, group B showed increased apoptosis. In group C, fetuin-A reduced the urea, creatinine, and phosphorus levels, and in group C, fetuin-A decreased inflammation and apoptosis by reduction of caspase-3 staining.

**Conclusion:**

Fetuin-A improved kidney function in CKD due to its anti-inflammatory and anti-fibrotic role.

## Introduction

Chronic kidney disease (CKD), also known as chronic kidney failure, is the gradual loss of kidney function. It may not become apparent in its early stages until it reaches an advanced stage, leading to the accumulation of fluid, electrolytes, and wastes and the development of anemia (1). CKD can progress to end-stage renal disease, which is fatal without artificial dialysis or a kidney transplant. Therefore, there is great urgency to slow the progression of renal damage by nonconventional, affordable therapy instead of expensive dialysis or kidney transplant (1). The adenine-induced CKD animal model has gained attention due to its simplicity of application without surgery. Orally administered adenine forms crystals in the proximal tubular epithelia, leading to tubulointerstitial fibrosis and inflammation that lead to kidney function impairment and anemia (2). Fetuin-A is a hepatokine, also known as alpha 2-Heremans-Schmid glycoprotein, with a half-life of 1-2 days and a molecular weight of approximately 60 kDa (3, 4). It is secreted mainly from the liver (>95%) (5). Fetuin-A is expressed in both renal tubular and glomerular cells (6). Fetuin-A is a multifunction protein as it inhibits a soft tissue calcification process (7), modulates innate immunity (8), and acts as a negative acute phase protein that decreases injury or infection (9). Serum fetuin-A is reduced in chronic renal disease (10, 11). These data are supported by Mohamed *et al*. (12), who studied the role of fetuin-A as an indicator of vascular disease in end-stage renal disease patients and correlations between fetuin-A and some kidney functions. They showed significant negative correlations between fetuin-A and creatinine, urea, and potassium. Additionally, a study (13) reported significant negative correlations between fetuin-A and creatinine. These data suggest that fetuin-A is related to kidney function and may play a role in chronic renal disease. Thus, this study aimed to investigate the effect of fetuin-A on renal functions in a CKD model.

## Materials and Methods

***Drugs and chemicals***

Fetuin-A from fetal bovine serum lyophilized powder (Sigma-Aldrich) and adenine (BioReagent, Sigma-Aldrich) were used.

***Experimental animals***

A total of 30 (local strain) adult male albino rats weighing 160-180 gm were obtained from the animal house of the Faculty of Veterinary Medicine, Zagazig University. The rats were acclimatized to animal house conditions for three weeks before conducting the experiments (14). The experimental protocol was approved by Physiology Department and by Zagazig University Institutional Animal Care Unit Committee (ZU-IACUC; Sharkia, Egypt) with approval number ZU-IACUC/3/F/137/2019. The animals were kept in plastic cages (30×18×24 inches). Every ten rats were housed per cage in the animal house of the Physiology Department, Faculty of Medicine, Zagazig University, under hygienic conditions. 

***Experimental protocol***

The study was conducted on 30 healthy adult male albino rats (10-12 weeks). After acclimatization for 3 weeks, the rats were divided into three equal groups (*n*=10 rats). Group A (the control group) was fed a normal diet for 21 days and then treated intraperitoneally with 0.5 ml saline every other day for 2 weeks. Group B (adenine-induced CKD group) was fed a normal diet with adenine 220 mg/kg daily taken orally for 21 days by oral gavages dissolved in sodium carboxymethyl cellulose 0.5% (15) and then treated intraperitoneally with 0.5 ml saline every other day for 2 weeks. Group C (fetuin-A-treated group) was fed a normal diet with adenine 220 mg/kg daily taken orally for 21 days by oral gavages dissolved in sodium carboxymethyl cellulose 0.5% (15) and intraperitoneally administered with fetuin-A 5 mg/kg (16) every other day for 2 weeks as the half-life of fetuin-A is 1-2 days (3, 4).

***Blood sampling and biochemical analysis***

Blood samples were collected 21 days after the induction of CKD to confirm impaired renal function in the adenine-induced CKD group and after injection of fetuin-A. Blood samples were collected from the orbital sinus veins in clean plastic centrifuge tubes. The samples were centrifuged at 5000 RMP for 15 min at 4 ^°^C. Then, the supernatant was collected and frozen at -70 ^°^C (17). Serum samples were assayed later for serum creatinine using enzyme-linked immunosorbent assay (ELISA) kits (18), serum urea using ELISA kits according to Kaplan and Glucose (19), serum sodium and serum potassium using ELISA kits according to Henry *et al.* (20), serum calcium using ELISA kits according to Robertson and Marshall (21), serum phosphorus using ELISA kits according to Bansal *et al.* (22), tumor necrosis factor (TNF) using the quantitative sandwich enzyme immunoassay technique according to Croft *et al.* (23) and Juhaz *et al. (*24), and interleukin-6 (IL-6) using ELISA kits according to Song *et al.* (25).

***Measuring estimated glomerular filtration rate (eGFR)***

eGFR was calculated using the following equations (26): 

Plasma creatinine ˂52 µmol/l: eGFR=880×W^0.695^×C^−0.660^× U^−0.391^, 

Plasma creatinine ≥52 µmol/l: eGFR=5862×W^0.695^×C^−1.150^× U^−0.391^.

***Immunohistochemistry (IHC)***

Kidney sections were stained immunohistochemically for caspase-3 expression (inactive caspase-3 (CPP32) Ab-4, rabbit polyclonal antibody (1:100 dilution), NeoMarker, Fremont, CA, USA) using a routine streptavidin-biotin-peroxidase technique according to the manufacturer’s recommendations (Rabbit and Mouse Specific HRP detection IHC kit (Ab93677), Abcam, Cambridge, UK). The color was improved using a 3,30-diaminobenzidine tetrahydrochloride substrate kit (ab64238-H_2_O_2_, Abcam, Cambridge, UK). Immunopositive reactions were revealed by the appearance of brown cytoplasmic staining analysis (27). 

***Quantitative analysis of caspase -3 staining***

‘0’ for negative staining to ‘1, 2, and 3’ for weak, moderate, and strong cytoplasmic staining, respectively (27). 

***Measurement of blood pressure***

Rat blood pressure was assessed at the beginning, after renal disease induction, and at the end of the experiment after injection of fetuin-A by a non-invasive blood pressure monitoring system (NIBP 250, Serial No.: 21202-108 BIOPAC System Inc., USA), which measures tail blood pressure by means of volume pressure (28, 29). Rats were put in a restrainer on a warmed platform, leaving their tails exposed outside the restrainer. Then, an occlusion cuff and a volume pressure-recording (VPR) sensor were placed near the tail base. With the slow deflation of the occlusion cuff, the tail blood flow returns, and then the VPR sensor measures the tail swelling that results from arterial pulsations from the blood flow. The digital value for the systolic blood pressure, diastolic blood pressure, and heart rate were recorded**.**

***Statistical analysis***

The data obtained were expressed as mean±standard deviation (SD) for quantitative variables and statistically analyzed using analysis of variance (ANOVA) F-test. Statistical analysis was performed using Statistical Package for Social Science version 25 (IBM, 25). A *P*-value less than 0.05 was considered statistically significant.

## Results

***Effect of adenine and fetuin-A on serum creatinine***

The adenine-induced CKD group showed a significant increase in serum creatinine compared with the control group (*P*<0.001), while the fetuin-A-treated group showed a significant decrease in serum creatinine compared with the adenine-induced CKD group (*P*<0.001) (Table 1).

***Effect of adenine and fetuin-A on serum urea***

The adenine-induced CKD group showed a significant increase in serum urea compared with the control group (*P*<0.001), while the fetuin-A-treated group showed a significant decrease in serum urea compared with the adenine-induced CKD group (*P*<0.001) (Table 1).

***Effect of adenine and fetuin-A on serum sodium***

The adenine-induced CKD group showed a significant decrease in serum sodium compared with the control group (*P*<0.001), while the fetuin-A-treated group showed a significant increase in serum sodium compared with the adenine-induced CKD group (*P*<0.001) (Table 1).

***Effect of adenine and fetuin-A on serum potassium***

The adenine-induced CKD group showed a significant increase in serum potassium compared with the control group (*P*<0.001), while the fetuin-A-treated group showed a significant decrease in serum potassium compared with the adenine-induced CKD group (*P*<0.01) (Table 1).

***Effect of adenine and fetuin-A on serum calcium***

The adenine-induced CKD group showed a significant decrease in serum calcium compared with the control group (*P*<0.001), while the fetuin-A-treated group showed an insignificant change in serum calcium compared with the adenine-induced CKD group (*P*> 0.05) (Table 1).

***Effect of adenine and fetuin-A on serum phosphorus***

The adenine-induced CKD group showed a significant increase in serum phosphorus compared with the control group (*P*<0.001), while the fetuin-A-treated group showed a significant decrease in serum phosphorus compared with the adenine-induced CKD group (*P*<0.01) (Table 1).

***Effect of adenine and fetuin-A on eGFR***

The adenine-induced CKD group showed a significant decrease in eGFR compared with the control group (*P*<0.001), while the fetuin-A-treated group showed a significant increase in eGFR compared with the adenine-induced CKD group (*P*<0.001) (Table 2).

***Effect of adenine and fetuin-A on mean arterial blood pressure (MABP)***

The adenine-induced CKD group showed a significant increase in MABP compared with the control group (*P*<0.001), while the fetuin-A group showed a significant decrease in MABP compared with the adenine-induced CKD group (*P*<0.001) (Table 2).

***Effect of adenine and fetuin-A on serum IL-6 and TNF***

**Figure 1:**
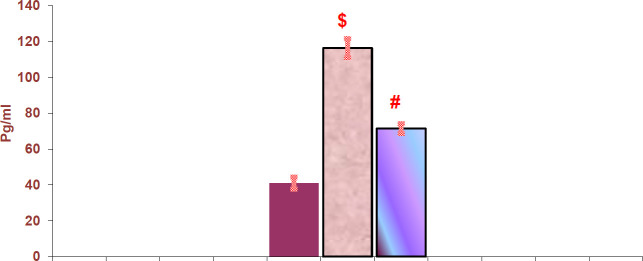
Histogram illustrates serum tumor necrosis factor (TNF)-α levels in all groups of rats

**Figure 2:**
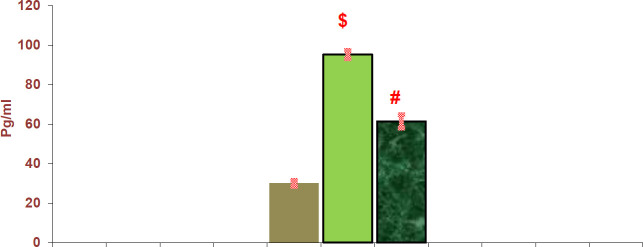
Histogram illustrates serum interleukin-6 (IL-6) levels in all groups of rats

The adenine-induced CKD group showed a significant increase in serum IL-6 and TNF-α compared with the control group (*P<*0.001), while the fetuin-A group showed a significant decrease in IL-6 and TNF compared with the adenine-induced CKD group (*P<*0.001) (Figures 1 and 2).

***Immunohistochemical study***

**Figure 3:**
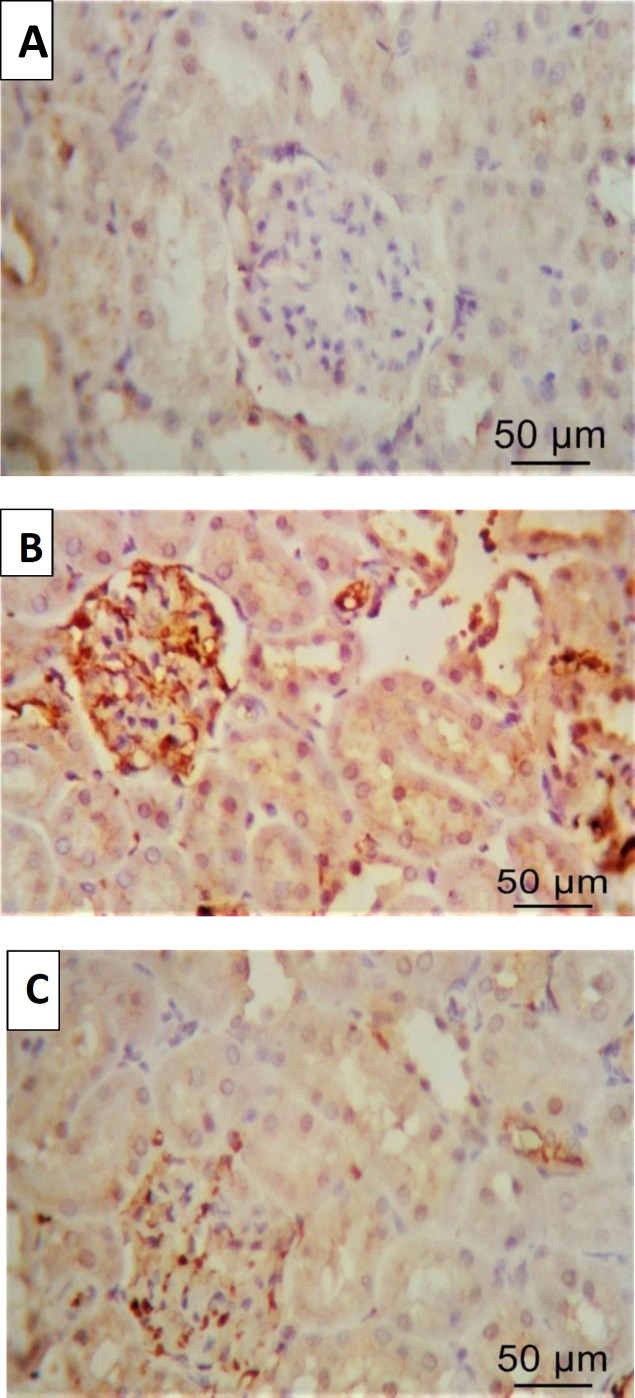
Immunohistochemical (IHC) study of rat kidney. (A) Cortical sections of the kidney tissue of group A with caspase-3 IHC showed mild cytoplasmic staining (400× magnification). (B) Cortical sections of the kidney tissue of group B with caspase-3 IHC showed marked diffuse cytoplasmic staining (400× magnification). (C) Cortical sections of the kidney tissue of group C with caspase-3 IHC showed moderate cytoplasmic staining (400× magnification)

Figure 3 shows the renal tissue section with caspase-3 IHC (400× magnification). Group A showed mild cytoplasmic staining (Figure 3A). Group B showed marked cytoplasmic staining (Figure 3B). Group C showed moderate cytoplasmic staining (Figure 3C).

***Quantitative analysis of caspase-3 staining in immunohistochemistry***

The adenine-induced CKD group showed a significant increase in caspase-3 cytoplasmic staining compared with the control group (*P*<0.001), while the fetuin-A group showed a significant decrease in caspase-3 cytoplasmic staining compared with the adenine-induced CKD group (*P*<0.001) (Table 3).

**Table 1:** Effect of oral adenine and fetuin-A on serum creatinine, urea, sodium, potassium, calcium, and phosphorus in all groups of rats

Groups		Group A	Group B	Group C
Parameters				
Serum creatinine (mg/dl)	$\overset{\text{®}}{X}$ ± SD	0.4 ± 0.027	2.13 ± 0.13	0.95 ± 0.07
	*p*-value of LSD		*P*<0.001^a^	*P*<0.001^b^
Serum urea (mg/dl)	$\overset{\text{®}}{X}$ ± SD	32.33 ± 2.31	116.8 ± 5.35	83.29 ± 3.96
	*p*-value of LSD		*P*<0.001^a^	*P*<0.001^b^
Serum sodium (mmol/L)	$\overset{\text{®}}{X}$ ± SD	144.04 ± 1.32	120.23 ± 1.83	132.08 ± 1.69
	*p*-value of LSD		*P*<0.001^a^	*P*<0.001^b^
Serum potassium (mmol/L)	$\overset{\text{®}}{X}$ ± SD	4.68 ± 0.19	7.63 ± 0.92	6.57 ± 0.93
	*p*-value of LSD		*P*<0.001^a^	*P*<0.01^b^
Serum calcium (mg/dL)	$\overset{\text{®}}{X}$ ± SD	10.8 ± 0.52	8.12 ± 0.6	8 ± 1
	*p*-value of LSD		*P*<0.001^a^	*p* > 0.05^b^
Serum phosphorus (mg/dL)	$\overset{\text{®}}{X}$ ± SD	5.85 ± 0.4141	9.19 ± 0.52	8.31 ± 0.71
	*p*-value of LSD		*P*<0.001^a^	*P*<0.01^b^

^a ^Significant compared with group A. ^b ^Significant compared with group B (I need more clarification, the groups were compared with either group A or group B); LSD, least significant difference

**Table 2:** Effect of oral adenine and fetuin-A on eGFR and MABP in all groups of rats

Groups		Group A	Group B	Group C
Parameters				
eGFR (ml/ min)	$\overset{\text{®}}{X}$ ± SD	1.6 ± 0.47	0.85 ± 0.5	1.13 ± 0.6
	*p*-value of LSD		*P*<0.001^a^	*P*<0.001^b^
MABP	$\overset{\text{®}}{X}$ ± SD	84.8 ± 4.44	132.8 ± 4.87	110.7 ± 4.97
	*p*-value of LSD		*P*<0.001^a^	*P*<0.001^b^

^a ^Significant compared with group A. ^b^ Significant compared with group B

eGFR: estimated glomerular filtration rate; MABP: mean arterial blood pressure

$= VS subgroup A; #=VS group B

$= VS subgroup A; #=VS group B

**Table 3:** Effect of oral adenine and fetuin-A on caspase-3 cytoplasmic staining in immunohistochemistry in all groups of rats

Group C	Group B	Group A	Quantitative analysis of caspase-3 staining in immunohistochemistry
1.4± 0.04	2. 85± 0.023	0.6± 0.05	±SD
*P*<0.001^b^	*P*<0.001^a^		P-value of LSD

^a ^Significant compared with group A. ^b^ Significant compared with group B

## Discussion

Fetuin-A is a hepatokine. It is secreted predominantly by the liver (>95%) (5). It is involved in essential physiological functions such as the regulation of acute inflammatory responses, bone mineralization (30), and calcium ion homeostasis and acts as a vascular calcification inhibitor (31). Fetuin-A is expressed in both renal tubular and glomerular cells (6), and its level is reduced in chronic renal disease (3, 32). Moreover, plasma fetuin-A levels progressively decrease with worsening renal function (13). This study was designed to investigate the possible effect of fetuin-A on some kidney functions in adenine-induced CKD in a male rat model. Regarding kidney function tests, this study showed that, in the adenine-induced CKD group, serum urea, creatinine, phosphorus, potassium, TNF, and IL-6 were high, and serum sodium and calcium were low. These data are consistent with those reported in other studies (33-36). Orally administered adenine is metabolized to 2, 8-dihydroxyadenine, which precipitates and crystallizes in proximal tubular epithelia, leading to the accumulation of crystals, thus causing degeneration of tubules, secondary inflammation, necrosis, and ultimately tubule-interstitial fibrosis that consequently produce renal dysfunction (33, 34). Additionally, in the adenine-induced CKD group, eGFR was low, which agrees with the result reported by Muñoz Abellán *et al*. (37). Moreover, in this study, IHC by caspase-3, which is a cysteine-aspartic acid protease that is best known for its apoptotic activity (38), showed increased apoptosis in the adenine-induced CKD group. This result is consistent with the results reported by Yang *et al.* (39), who induced CKD by subtotal nephrectomy and reported a progressive increase in apoptosis of tubular and interstitial cells, thus contributing to tubular atrophy and the associated renal fibrosis. In group C (the fetuin-A-treated group), we observed that fetuin-A improved kidney function by reducing the urea, creatinine, potassium, and phosphorus levels and increasing the sodium levels. These data are supported by Mohamed *et al*. (12), who studied the role of fetuin-A as an indicator of vascular disease in end-stage renal disease patients and correlations between fetuin-A and some kidney functions. They reported significant negative correlations between fetuin-A and creatinine, urea, and potassium. Furthermore, Multuay *et al*. (13) reported significant negative correlations between fetuin-A and creatinine. Additionally, in group C, fetuin-A increased eGFR. Fetuin-A may antagonize the adenine inflammatory effect, as it reduced the TNF and IL-6 levels. These findings are consistent with those reported by researchers (40) who reported that fetuin-A plays a role in inflammation by down-regulating the pro-inflammatory cytokines produced by macrophages (41, 42), plays a role in macrophage deactivation, and plays an anti-inflammatory role (42). Treatment with fetuin-A reduced kidney injury and caspase-3 staining in group C, fetuin-A may play a role in inhibiting renal fibrosis as it has an anti-fibrotic effect (43, 44), and fetuin-A acts as an antagonist of the transforming growth factor. It could be demonstrated that the incubation of fetuin-A with hepatic stellate cells significantly inhibited collagen synthesis in hepatic stellate cells, potentially linking fetuin-A as an anti-fibrotic agent (43). The adenine-induced CKD group showed higher MABP than the control group. Increased MABP as a result of increased renin-angiotensin system activity (45) in CKD leads to vasoconstriction and aldosterone secretion that leads to sodium retention, increased blood pressure (46), and tubular damage (47) that leads to more sodium retention and more elevation in blood pressure (48). Additionally, vascular calcification that occurs in CKD (49) leads to the elevation of blood pressure (50) by decreasing the elasticity of the vessels, which results in reduced vascular compliance (51). We observed in group C that fetuin-A reduced MABP, and this may be due to the improvement in kidney function that leads to a decreased probability of hypertension development and the inhibitory effect of fetuin-A on vascular calcification (52) as lower levels of fetuin-A were associated with higher vascular calcification in patients not yet on renal replacement therapy and patients with end-stage renal disease (13). Fetuin-A interacts with calcium and phosphate, allowing the formation of calciprotein monomers that are eliminated by the reticuloendothelial system, which prevents vascular deposition and calcification (53). Our results are consistent with those reported by Cuspidi and Sala (54), who showed that plasma fetuin-A concentrations were low in hypertensive subjects. Furthermore, low circulating fetuin-A concentrations have been evidenced during progressive aortic stiffening and calcification in the course of some kidney pathologies (3, 55, 56).

## Conclusion

The study results showed that fetuin-A improved kidney functions, and this may be due to its anti-inflammato rmore, it reduced arterial blood pressure, and this may be due to its inhibitory effect on vascular calcification. Further studies are needed to evaluate the effects of fetuin-A on renal function in acute renal failure.

## Authors’ Contributions

WR, MZ, HE, and RE designed the experiments; HE performed experiments and collected data; WR, MZ, and RE discussed the results and strategy; RE supervised, directed, and managed the study; WR, MZ, HE, and RE approved the final version to be published.

## Data availability statement

The datasets used and analyzed during the current study are available from the corresponding author upon reasonable request.

## Conflicts of Interest

The authors declare that there are no conflicts of interest regarding the publication of this paper.
